# Effect of Mg-Gluconate on the Osmotic Fragility of Red Blood Cells, Lipid Peroxidation, and Ca^2+^-ATPase (PMCA) Activity of Placental Homogenates and Red Blood Cell Ghosts From Salt-Loaded Pregnant Rats

**DOI:** 10.3389/fphys.2022.794572

**Published:** 2022-01-27

**Authors:** Deliana Rojas, Cilia Abad, Sandy Piñero, Yollyseth Medina, Delia I. Chiarello, Fulgencio Proverbio, Reinaldo Marín

**Affiliations:** Center for Biophysics and Biochemistry (CBB), Venezuelan Institute for Scientific Research (IVIC), Caracas, Venezuela

**Keywords:** magnesium gluconate, PMCA, TBARS, preeclampsia, salt-loaded pregnant rats, osmotic fragility

## Abstract

Preeclampsia (PE) is a pregnancy-specific syndrome with multisystem involvement which leads to fetal, neonatal, and maternal morbidity and mortality. A model of salt-loaded pregnant rats has been previously studied, sharing several pathological characteristics of preeclamptic women. In this study, it was compared the effects of the treatment with an oral magnesium salt, magnesium gluconate (Mg-gluconate), on the osmotic fragility of red blood cells, lipid peroxidation, and PMCA activity of placental homogenates and red blood cell ghosts in salt-loaded pregnant rats. Mg-gluconate has a higher antioxidant capacity than MgSO_4_ due to the presence of several hydroxyl groups in the two anions of this salt. Salt-loaded pregnant rats received 1.8% NaCl solution *ad libitum* as a beverage during the last week of pregnancy. On day 22nd of pregnancy, the rats were euthanized and red blood cells and placenta were obtained. Salt-loaded pregnant rats showed an increased level of lipid peroxidation and a lowered PMCA activity in placental and red blood cell ghosts, as well as an increased osmotic fragility of their red blood cells. The treatment of the salt-loaded pregnant rats with Mg-gluconate avoids the rise in the level of lipid peroxidation and the concomitant lowering of the PMCA activity of their red blood cell membranes, reaching values similar to those from control pregnant rats. Also, this treatment prevents the increase of the osmotic fragility of their red blood cells, keeping values similar to those from control pregnant rats. Mg-gluconate seems to be an important candidate for the replacement of the MgSO_4_ treatment of preeclamptic women.

## Introduction

Preeclampsia (PE) is a complex, pregnancy-related multisystemic disorder that annually affects an estimated 8.5 million pregnant women worldwide (Burton et al., 2019). This syndrome is associated with substantial maternal and fetal morbidity and mortality, complicating 2–8% of pregnancies worldwide (Steegers et al., 2010). Current guidelines from the International Society for the Study of Hypertension in Pregnancy (ISSHP) define PE as a new-onset of hypertension and the coexistence of one or more of the following new-onset conditions: proteinuria (≥300 mg/day), maternal organ dysfunction, such as acute renal insufficiency, liver, neurological, or hematological complications, and/or uteroplacental dysfunction (including fetal growth restriction and intrauterine death; Brown et al., 2018). The precise mechanisms underlying the cause(s) of PE are still largely unknown; nevertheless, the consensus is that PE is triggered either by specific trophoblast defects (placental origin), recognized as early-onset PE (eoPE), or by maternal metabolic defects (maternal origin), recognized as late-onset PE (loPE). eoPE is characterized by abnormal placentation due to defective spiral artery remodeling and abnormal cytotrophoblast invasion thereby causing resistance to blood flow in the uterine arteries, placental hypoperfusion, and hypoxia. loPE occurs at the later stages of pregnancy and might be due to the interaction between a healthy placenta and maternal factors that would ultimately cause microvascular damage and maternal endothelial dysfunction (Nirupama et al., 2021). Within the pathophysiological events, associated with both subtypes of PE, increased oxidative stress and systemic inflammatory response are included (Chiarello et al., 2020). Oxidative stress biomarkers have been described in red blood cells, blood plasma, and placental tissue of women with PE during the last 50 years (Roberts and Cooper, 2001; Madazli et al., 2002; Takacs et al., 2003). Moreover, the severity of the disease is related to the level of lipid peroxidation both in serum (Serdar et al., 2006) and red blood cells (Madazli et al., 1999).

Despite extensive research on PE during the last 20 years, there is no single treatment for PE except delivery of the baby (removal of the placenta), which poses a significant risk to both mother and baby. Nevertheless, therapies to attenuate symptoms and maintain pregnancy are necessary to prolong gestation and allow fetal growth and maturation. This absence of effective therapies makes the animal models of PE essential to identify causal pathways, develop new therapies, and evaluate the safety and efficacy of treatments before evaluation in clinical trials. Despite the spontaneous development of PE, this clinical syndrome is essentially limited to the human species, there have been reported several experimental animal models of PE (Bakrania et al., 2020). One of them consists in feeding a 1.8% NaCl solution to pregnant rats *via* oral during the last week of gestation as replacement of drinking water (Beauséjour et al., 2003, 2007a,b). As shown in Table 1, during this treatment, the pregnant rats exhibit many of the clinical features of human PE, such as increased systolic blood pressure, proteinuria, increased oxidative and nitrosative stress, decreased intrauterine growth, decreased activity of the renin-angiotensin-aldosterone system, increased thromboxane/prostacyclin ratio, and reduced diameter of uterine arcuate artery segment and placental weights. Moreover, similar to preeclamptic women, pregnant salt-loaded rats experience a reversal of the blunted responses to vasoconstrictors associated with normal pregnancy (Auger et al., 2004). These features have been interpreted as an indication of a decreased placental perfusion in the salt-loaded pregnant rats (Beauséjour et al., 2003; St-Louis et al., 2006). In our laboratory, we have shown that blood plasma, red blood cells ghosts, and placental homogenates of salt-loaded pregnant rats show an increased level of lipid peroxidation, as well as a concomitant, lowered PMCA activity (Rojas et al., 2015) which was previously shown in both red blood cell ghosts and syncytiotrophoblast plasma membranes from preeclamptic pregnant women (Matteo et al., 1998; Abad et al., 2012). Additionally, red blood cells from experimental rats displayed higher osmotic fragility compare to control rats. Interestingly, similar results have been described in preeclamptic women (Abad et al., 2005, 2010, 2015b).

**Table 1:** Comparison of several parameters between preeclamptic women and salt-loaded pregnant rats.

Parameter	Preeclamptic women	Salt-loaded pregnant rats
Systolic blood pressure	↑ Eiland et al., 2012; Abad et al., 2015a	↑ Beauséjour et al., 2003
Proteinuria	↑ Eiland et al., 2012; Abad et al., 2015a	↑ Beauséjour et al., 2003
Oxidative stress	↑ Chiarello et al., 2020	↑ Beauséjour et al., 2007a,b; Rojas et al., 2015
Nitrosative stress	↑ Tashie et al., 2020	↑ Beauséjour et al., 2007b
IUGR	↑ Sibai, 2003	↑ Rojas et al., 2015
Uterine arcuate artery function	Abnormal; Gyselaers et al., 2014; Ives et al., 2020	Abnormal; St-Louis et al., 2006
RAAS	Dysfunctional; Lumbers et al., 2019	Dysfunctional; Beauséjour et al., 2003
TxB_2_/6-keto-PGF_1α_ ratio	↑ Dekker and Sibai, 1998	↑ Beauséjour et al., 2007a
Placental TBARS	↑ Casart et al., 2001; Abad et al., 2005	↑ Rojas et al., 2015
RBCG TBARS	↑ Carreiras et al., 2002; Abad et al., 2005	↑ Rojas et al., 2015
Placental PMCA	↓ Casart et al., 2001; Abad et al., 2005	↓ Rojas et al., 2015
RBCG PMCA	↓ Carreiras et al., 2002; Abad et al., 2005	↓ Rojas et al., 2015
RBC osmotic fragility	↑ Abad et al., 2010, 2012	↑ Rojas et al., 2015

The current knowledge on the role of magnesium treatment in PE with severe features has focused on the study of magnesium sulfate (MgSO_4_; Amaral et al., 2017; Chiarello et al., 2018). MgSO_4_ is an important pharmacological tool used to treat PE with severe features because it can prevent the recurrent seizures of eclampsia and tocolysis in preterm labor (Sibai, 2005; Smith et al., 2013; Da Costa et al., 2020). This salt is mainly administered intravenously because the oral route is well known to produce gastrointestinal effects and it is poorly absorbed in the gut (Lu and Nightingale, 2000). Although MgSO_4_ has been used for many years, its mechanism of action at the molecular level in preeclamptic/eclamptic pregnant women remains an enigma. In recent studies, important advances in computational chemistry have shed some light on its mechanisms of action (Fernández et al., 2017, 2021).

It is important to mention that clinical use of MgSO_4_ should be done in the hospital, as it presents significant toxicity risks. The clinical effect and toxicity of MgSO_4_ are linked to its concentration in blood plasma. While a plasma concentration of 1.8 to 3.0 mmol/L has been suggested for treatment of eclamptic convulsions, the actual magnesium dose and concentration needed for prophylaxis have never been estimated (Lu and Nightingale, 2000). Hence, this would justify trying to obtain an oral Mg salt as effective or more effective than MgSO_4_ for preeclamptic patients with severe features. Oral salts of Mg can be absorbed intestinally with little or no gastrointestinal effects. In this regard, there is a study showing that orally ingested magnesium, as Mg-gluconate, can significantly increase serum magnesium levels (Martin et al., 1987). Furthermore, Mg-gluconate used as an oral tocolytic is as effective as a beta-agonist in patients whose labor is arrested initially with intravenous therapy (Martin et al., 1988).

**Figure 1:**
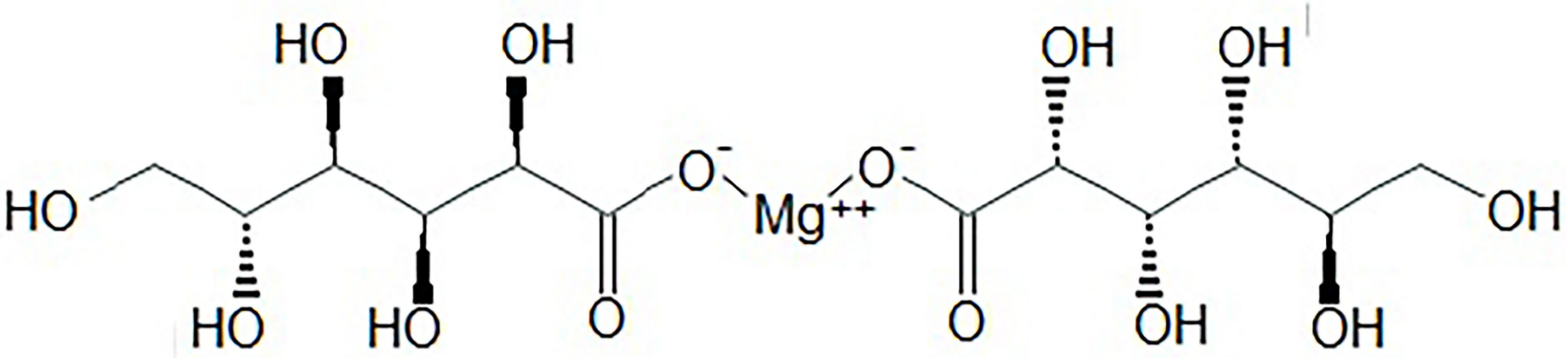
Molecular structure of Mg-gluconate.

In the current study, we decided to use an oral magnesium salt, such as Mg-gluconate, which has a higher antioxidant capacity than MgSO_4_ (Mak et al., 2000), due to the presence of several hydroxyl groups in the two anions of this salt (Figure 1). In this regard, Mg-gluconate, dose-dependently reduced oxidative damage (decreased TBARS) in an isolated membrane preparation exposed to exogenous free radicals (Mak et al., 2000). Mg-gluconate increased the survival and proliferation of cultured endothelial cells, as well as reduced the loss of exogenous glutathione caused by increased oxidative stress. When comparing the effects of similar concentrations of MgSO_4_ and MgCl_2_, these salts were about 60% less effective than Mg-gluconate (Mak et al., 2000). Mg-gluconate has been used in obstetrics as a tocolytic agent in threatened abortion and preterm labor without major side effects (Pérez et al., 1949; Martin et al., 1987, 1998). In addition, it has been suggested that Mg-gluconate may have additional therapeutic benefits for clinical use in those pathologies associated with increased levels of free radicals (Mak et al., 2000).

This study aims to evaluate the effect of an oral salt of magnesium, magnesium gluconate (Figure 1, MgC_12_H_22_O_14_, in this study as Mg-gluconate), in salt-loaded pregnant rats as an animal model of PE. In the current study, it was analyzed the effect of this treatment, on the osmotic fragility of red blood cells, lipid peroxidation, and PMCA activity in both red blood cells and placental homogenates.

## Materials and Methods

### Animal Welfare

All animal experiments were performed with the approval of the Ethics Committee for Animal Experiments of the Instituto Venezolano de Investigaciones Científicas, IVIC (Cobianim). A total of 100 female Sprague–Dawley rats (bodyweight 225–250 g, 3 months old) were used in the experiments. All efforts were made to minimize the number of animals used.

### Experimental Design

Female Sprague–Dawley rats were randomly divided into two groups: the first group (group to mate with a male for fertilization) and the second group (non-pregnant group). All groups of virgin female rats were housed, four animals per cage, with constant room temperature (23 ± 1°C, mean ± SD), low noise, and the following light–darkness schedule: 12 h of light (06:00–18:00 h, 80 lux) and 12 h of darkness (18:00–06:00 h). All the rats received food and water *ad libitum*. The virgin female rats of the first group were divided as follows: control non-pregnant rats that had tap water during 1 week (CNP); control non-pregnant rats had tap water with Mg-gluconate during 1 week (CNP+Mg-gluconate); salt-loaded non-pregnant rats were kept drinking a solution of 1.8% NaCl (SLNP); and salt-loaded non-pregnant rats were kept drinking a solution of 1.8% NaCl with Mg-gluconate during 1 week (SLNP+Mg-gluconate). This group was used to standardize the Mg-gluconate treatment. The virgin female rats of the second group were bed with a known fertile male in the animal room. Day 1 of pregnancy was established when spermatozoa were found in morning vaginal smears. On day 15 of pregnancy, pregnant rats were randomized into four groups: control pregnant rats that had tap water during the last week of pregnancy (CP); control pregnant rats that had tap water with Mg-gluconate during the last week of pregnancy (CP+Mg-gluconate); salt-loaded pregnant rats were kept drinking a solution of 1.8% NaCl during the last week of their pregnancy (SLP); and salt-loaded pregnant rats were kept drinking a solution of 1.8% NaCl with Mg-gluconate during the last week of their pregnancy (SLP+Mg-gluconate). All pregnant female rats were weighed during the last week of their pregnancy. At the end of the treatment (22nd day of pregnancy), the rats were i.p. anesthetized with thiopental and euthanized. Immediately, 5–10 ml of blood were drawn from the left ventricle utilizing heparinized syringes, and then, in the pregnant group, the placentas and fetuses were removed. The fetuses and the placentas were then weighted and measured. The fetuses were then euthanized. The placentas were kept in an ice-cold 0.9% NaCl solution until use.

### Blood Pressure Measurements

Blood pressure of pregnant groups was routinely measured by the indirect tail-cuff method (MRBP Mouse and Rat Tail Cuff Method Blood Pressure Systems, IITC Life Science Inc., CA, United States) from the first week of pregnancy until the 19th day of pregnancy. On the 19th day of pregnancy, the rats were housed in metabolic cages until the end of the treatment (22nd day of pregnancy).

### Determination of Serum Magnesium

QuantiChrom™ assay kit for serum magnesium determination was purchased from BioAssay Systems (CA, United States). The serum magnesium concentration was measured colorimetrically using the calmagite complexometric method following the kit manufacturer’s instructions.

### Evaluation of Protein in the Urine

The 24 h urine collected was centrifuged at 16,000 × *g* for 10 min and diluted 10× with distilled water and assayed for protein content (Bradford, 1976).

### Preparation and Homogenization of Rat Placentas

The placentas, without the umbilical cord, were washed with 0.9% NaCl at 4°C. After weighing and cleaning, the amniochorion and the chorionic plate were removed, and the placentas were cut in small pieces and homogenized (3 ml/g) at 4°C in one of the following solutions: (a) 250 mm sucrose; 50 mm Tris-Hepes, pH 7.2; 5 mm EGTA; 5 mm EDTA; and 1 mm phenylmethylsulfonyl fluoride (PMSF; buffer 1) and (b) 5 mm dibasic phosphate (Na_2_HPO_4_; pH 7.4), 150 mm NaCl, 1 mm PMSF (buffer 2). The homogenates prepared with buffer 1 were used for PMCA activity assays, while the homogenates prepared with buffer 2 were used for Thiobarbituric Acid Reactive Substances (TBARS) assay. The homogenates were filtered through gauze filters and stored in the freezer at −70°C until use, for no longer than 7 days.

### Osmotic Fragility of Red Blood Cells

The osmotic fragility of red blood cells was determined according to a method described elsewhere (Abad et al., 2010). The osmotic fragility curves were fitted with the Boltzmann equation for sigmoidal fitting, using the software Origin^®^ (OriginLab Corporation, United States). The osmolar concentrations producing 50% hemolysis of the added red blood cells were defined as OS_50_, and it was calculated from the hemolysis curves and used as a parameter of osmotic fragility. All the determinations were run in quadruplicate.

### Red Blood Cell Ghosts

Heparinized blood samples were centrifuged at 12,000 × *g*, 5 min, 4°C. The blood plasma was saved, the buffy coat was discarded, and the packed red blood cells were washed three times by centrifugation under the same conditions, in a solution containing 150 mm NaCl and 10 mm Tris–HCl (pH 7.5 at 0°C). The washed erythrocytes were then utilized to prepare hemoglobin-free red blood cell ghosts, according to the method of Heinz and Hoffman (1965). The ghosts were stored in a solution containing 17 mm Tris–HCl and 0.1 mm EDTA (pH 7.5 at 0°C) and kept frozen at −70°C until use.

### PMCA Assay

PMCA activity of placental homogenates was determined by a modification of the method previously described (Borrego et al., 2006). The amount of inorganic phosphate liberated from the hydrolysis of ATP was determined as described elsewhere (Marín et al., 1986). The assay was carried out in the presence of 1 mm thapsigargin, with and without 756 μm free Ca^2+^. The PMCA activity is expressed as nmol Pi/mg protein min, after subtraction of a blank run in parallel under the same conditions. The protein concentration, in all the cases, was determined according to the method of Bradford (1976). The thapsigargin-insensitive PMCA activity was calculated as the difference in the phosphate liberated in a medium containing Mg^2+^+Ca^2+^ minus the one liberated in the same medium, but in the absence of Ca^2+^. To avoid the presence of membrane vesicles, the different fractions were always pretreated before the assays with sodium dodecyl sulfate (SDS), as previously described (Proverbio et al., 1986), at a ratio of 1.25 SDS/protein.

The ATPase activity of the red blood cell ghosts was similarly determined by measuring the quantity of inorganic phosphate liberated from the hydrolysis of ATP, following the ethod described by Nardulli et al. (1994). The liberated inorganic phosphorus was determined in an ELISA (Tecan, San Jose, CA – Sunrise) spectrophotometer at 705 nm, following the Fiske–Subbarow method (Fiske and Subbarow, 1925).

### Lipid Peroxidation Measurements

The amount of lipid peroxidation of the red cell ghosts and placental homogenates was estimated by measuring TBARS following a method described by Feix et al. (1991). The absorbance was measured at 532 nm, and the TBARS values were calculated using the malondialdehyde (MDA) standard curve, prepared by acid hydrolysis of 1,1,3,3-Tetramethoxypropane. The values are expressed as nmoles of MDA per milligram of protein.

### Statistical Analysis

The statistical analysis was performed with the software InStat (GraphPad Software, United States). Comparisons between treatment conditions were assessed by one-way ANOVA with the *post-hoc* analysis with the Student–Newman–Keuls test. Statistical analysis between two conditions was performed by the Student’s *t*-test. All results were expressed as means ± SE, and *n* represents the number of experiments performed with different preparations. In all cases, the PMCA activity was calculated by paired data. The comparison scheme that was used in this study was the following:

1. CP vs. CP+Mg-gluconate; in order to test if Mg-gluconate treatment interferes with basal condition.
2. CP vs. SLP; in order to test if the sodium supplementation treatment indeed induces alterations.
3. SLP vs. SLP+Mg-gluconate; in order to test if Mg-gluconate prevents the negative effect of sodium supplementation treatment.

For this comparison scheme, value of *p* 0.02 was accepted as statistically significant.

## Results

The oral dose of Mg-gluconate necessary to raise serum magnesium levels was determined in rats treated or not with a solution of 1.8% NaCl. Daily oral doses of Mg-gluconate between 1.97 and 2.70 g/kg body weight significantly raised serum magnesium levels in both control non-pregnant (CNP) and salt-loaded non-pregnant rats (SLNP; Supplementary Table 1). The increase in serum magnesium levels with the oral doses of Mg-gluconate was independent of rat pregnancy and salt overload (Supplementary Table 2).

During sodium supplementation (1.8%; days 15–22), there was an increase in water and sodium intake in these pregnant rats, together with a significant reduction in food intake and body weight variation between days 15 and 22 of gestation (Supplementary Table 3). The addition of Mg-gluconate to the drinking water of control pregnant rats (CP+Mg-gluconate) did not affect either water intake, food intake, or bodyweight variation during the treatment time (Supplementary Table 3). In contrast, in salt-loaded pregnant rats, the presence of Mg-gluconate in the drinking water (SLP+Mg-gluconate) reduced fluid intake and consequently also reduced sodium intake. Despite the increase in food intake, these pregnant rats presented the lowest body weight variation (Supplementary Table 3). In this particular, it should be mentioned that the high doses of Mg-gluconate in the SLP+Mg-gluconate group produced an increase in gastrointestinal transit with the presence of watery feces, which could explain the reduction in body weight variation in this group of pregnant rats.

The variation in body weight of the rats during gestation may well have an important impact on fetal parameters. For this purpose, the placental and fetal weights, and the number of fetuses per litter were determined for the different conditions under study. Table 2 shows the results obtained. Notice that as previously reported (Beauséjour et al., 2003; Rojas et al., 2015), the increase of sodium intake in pregnant rats (SLP) was accompanied by a significant decrease in placental and fetal weight. The presence of Mg-gluconate in the drinking water of control pregnant rats did not produce any significant change in the fetal parameters of the rats. In contrast, in the case of salt-loaded pregnant rats, it prevented the decrease in placental and fetal weights characteristic of pregnant rats with high sodium intake.

**Table 2:** Effects of the treatment with Mg-gluconate on fetal parameters in control (CP) and salt-loaded pregnant rats (SLP).

Parameter	CP	CP + Mg-gluconate	SLP	SLP + Mg-gluconate
Placental weight (g)	0.51 ± 0.01 (110) [9]	0.51 ± 0.02 (35) [3]	0.45 ± 0.01 (105) [9]^a^	0.51 ± 0.02 (75) [6]
Fetal weight (g)	5.44 ± 0.13 (110) [9]	5.53 ± 0.10 (35) [3]	4.48 ± 0.16 (105) [9]^b^	5.37 ± 0.13 (75) [6]
Number of fetuses/rat	12 ± 1 [9]	12 ± 2 [3]	12 ± 1 [9]	13 ± 1 [6]

*Values in parentheses indicate the total number of fetuses studied. Values in brackets indicate the total number of mothers studied in each case. Values expressed as the mean ± S.E. Comparisons between treatment conditions were assessed by one-way ANOVA with the post-hoc analysis with the Student–Newman–Keuls test*. *For both placental and fetal weights, value of p for the ANOVA test was <0.001*.

a *p < 0.001 vs. CP; p < 0.01 vs. SLP + Mg-gluconate*.

b *p < 0.001 vs. CP, vs. SLP + Mg-gluconate*.

Sodium supplementation (1.8%) during the last week of gestation produced a significant increase in blood pressure compared to the values of control pregnant rats (Table 3), which is consistent with what has been reported previously (Beauséjour et al., 2003). Significantly, the presence of Mg-gluconate during the salt overload of pregnant rats prevented the increase in blood pressure and brought it to values similar to those of control pregnant rats. Additionally, the salt overload of pregnant rats produced significant proteinuria, which was prevented by the presence of Mg-gluconate during sodium supplementation (Table 3).

**Table 3:** Effects of the treatment with Mg-gluconate on blood pressure and urinary protein excretion in control (CP) and salt-loaded pregnant rats (SLP).

Parameter	CP (*n* = 7)	CP+Mg-gluconate (*n* = 7)	SLP (*n* = 10)	SLP+Mg-gluconate (*n* = 10)
Systolic blood pressure (mmHg)	102.89 ± 1.46	105.48 ± 1.42	122.00 ± 1.22^a^	107.09 ± 0.97
Diastolic blood pressure (mmHg)	65.06 ± 1.70	64.59 ± 2.83	78.81 ± 2.45^b^	58.35 ± 3.11
Mean blood Pressure (mmHg)	79.06 ± 1.70	80.12 ± 1.97	93.38 ± 2.26^a^	74.11 ± 2.11
Protein excretion (mg/24 h)	5.97 ± 0.65	5.44 ± 1.11	11.92 ± 0.88^a^	5.35 ± 0.74

*The data for blood pressure in this table corresponds to the 19th day of pregnancy. The 24 h urine was collected on the 21st day of pregnancy. Comparisons between treatment conditions were assessed by one-way ANOVA with the post-hoc analysis with the Student–Newman–Keuls test. In each row, value of p for the ANOVA test was <0.001*.

a *p < 0.001* vs. *CP, vs. SLP + Mg-gluconate*.

b *p < 0.01 vs. CP, <0.001 vs. SLP + Mg-gluconate*.

**Figure 2:**
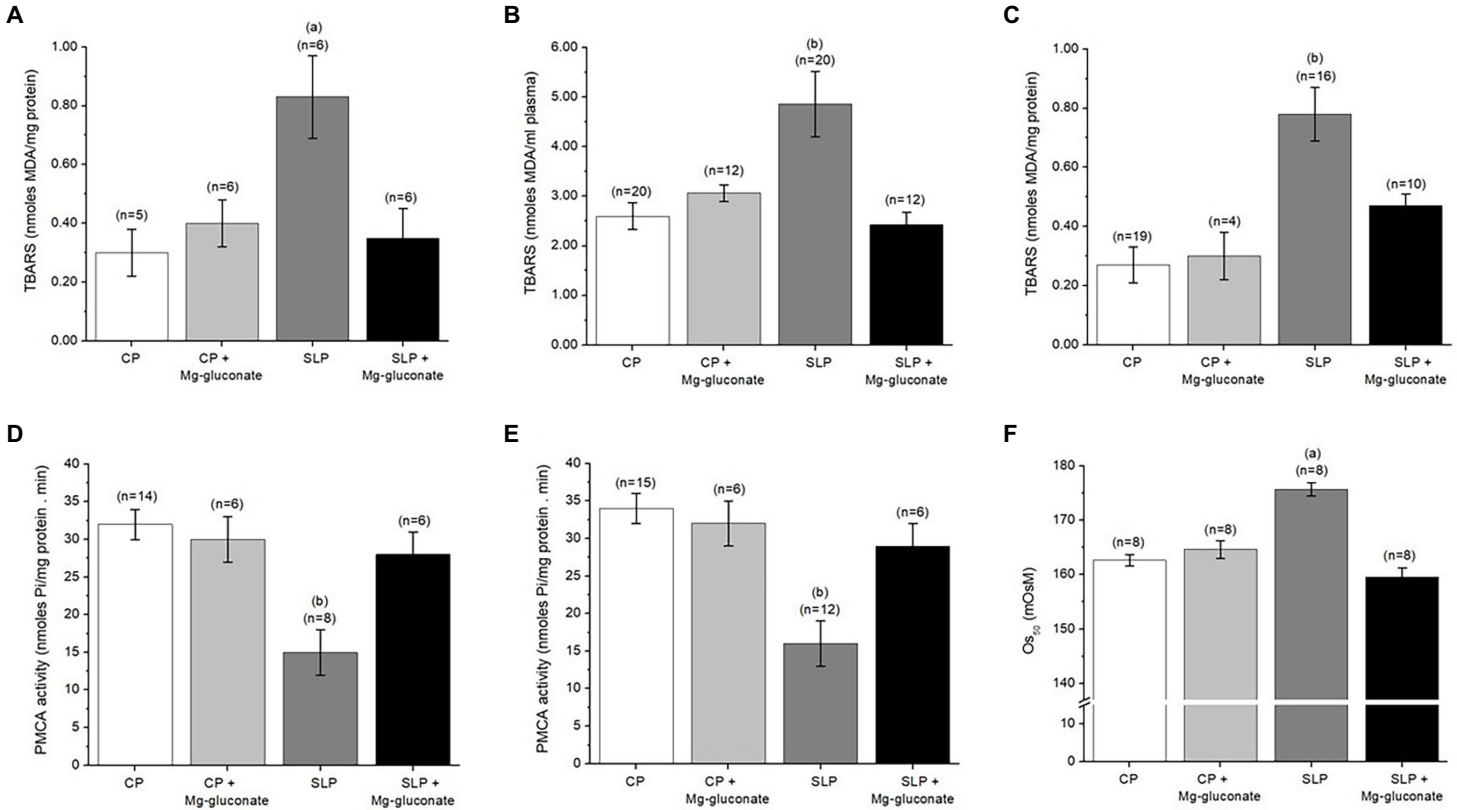
Mg-gluconate alleviated the level of oxidative stress, the reduction of the PMCA activity, and the osmotic fragility of red blood cells produced by salt overloading of pregnant rats during the last week of pregnancy. **(A-C)** Effect of the treatment with Mg-gluconate on TBARS in red cell ghosts **(A)**, blood plasma **(B)**, and placental homogenates **(C)** from control (CP) and salt-loaded pregnant rats (SLP). **(D,E)** Effect of the treatment with Mg-gluconate on PMCA activity in red cell ghosts **(D)** and placental homogenates **(E)** from CP and SLP. **(F)** Effect of treatment with Mg-gluconate on the osmotic fragility (OS_50_) of intact red blood cells from CP and SLP. OS_50_ is defined as the osmolar concentration that produces 50% hemolysis of the added red blood cells. The lysis was performed as indicated in the Materials and Methods section. Pregnant female Sprague–Dawley rats (bodyweight 225–250 g, 3 months old) were kept drinking either tap water (CP) or a solution of 1.8% NaCl (SLP), during the last week of their pregnancy, with and without Mg-gluconate in the drinking solution. Values are means ± S.E. Comparisons between treatment conditions were assessed by one-way ANOVA with the *post-hoc* analysis with the Student–Newman–Keuls test. Value of *p* for the ANOVA test was <0.01 (Panel **A**) and < 0.001 (Panels **B–F**). (a) *p* < 0.01 vs. CP, vs. SLP+Mg-gluconate. (b) *p* < 0.001 vs. CP, *p* < 0.01 vs. SLP+Mg-gluconate.

It is well known that there is a close relationship between oxidative stress and PE, both in eoPE and loPE subtypes (Madazli et al., 1999, 2002; Roberts and Cooper, 2001; Takacs et al., 2003; Chiarello et al., 2020). Additionally, in the animal model of PE that we used in the present study, an increase in oxidative stress at the placental, kidney, and heart level has been demonstrated (Beauséjour et al., 2007a,b). Consequently, it was decided to evaluate the effect of Mg-gluconate treatment on TBARS levels in the placenta, blood plasma, and red blood cells of sodium-overloaded pregnant rats. Figures 2A–C show the results of this study. Notice that sodium overload in pregnant rats produces a significant increase in the levels of TBARS in the placenta, blood plasma, and red blood cell ghosts. The presence of Mg-gluconate during sodium overload in pregnant rats prevents the increase in TBARS levels in these animals.

It has been shown that there is an inversely proportional relationship between increased oxidative stress on plasma membranes and reduced activity of several membrane enzymes that have a strong dependence on the lipid environment, such as PMCA (Matteo et al., 1998; López et al., 2003). In this particular, since during PE, there is an increase in lipid peroxidation of plasma membranes, our group has shown that associated with PE there is a reduction in PMCA activity, which does not seem to be related to a reduction in the number of PMCA molecules. Therefore, we evaluated the effect of the presence of Mg-gluconate during sodium overload in pregnant rats on PMCA activity in both placental homogenates and red blood cell ghosts. The results of this study are presented in Figures 2D,E. It can be seen that sodium supplementation in pregnant rats causes a significant decrease in PMCA activity in the placenta and RBC ghosts. This is in agreement with our previous studies (Rojas et al., 2015). The presence of Mg-gluconate during sodium supplementation prevents the decrease in PMCA activity.

In a previous study, we have found that sodium supplementation of pregnant rats during the last week of gestation causes an increased sensitivity of intact red blood cells to osmotic shock, also known as osmotic fragility (Rojas et al., 2015). In the present study, the effect of Mg-gluconate on osmotic fragility in pregnant rats with or without salt overload was analyzed by using the osmotic fragility curves (Supplementary Figure 1). With these curves, the OS_50_ was calculated and the values are presented in Figure 2F. It can be observed that Mg-gluconate treatment of pregnant rats subjected to sodium supplementation has a significant impact on the OS_50_ of intact red blood cells, bringing these values to those of pregnant control rats.

## Discussion

In this study, it was found that the presence of Mg-gluconate during salt overload of pregnant rats blocks the deleterious effects of sodium supplementation during the last week of gestation. In this particular, it is important to highlight the positive effect on blood pressure (Table 3); proteinuria (Table 3); TBARS in red blood cells, blood plasma, and placenta (Figures 2A–C); PMCA activity in red cell ghosts and placenta (Figures 2D,E), and osmotic fragility of intact red blood cells (Figure 2F). These effects of Mg-gluconate treatment in the animal model of PE that we used are of great importance for the treatment of this pathology. It is important to mention that this study did not include an experimental group with MgSO_4_ in order to compare it with Mg-gluconate. The use of MgSO_4_ has the main disadvantage that it is mainly administered intravenously because the oral route is well known to produce gastrointestinal effects (Lu and Nightingale, 2000).

The effect of Mg-gluconate on blood pressure in salt-loaded pregnant rats highlights a process involving the complex mechanisms that control blood pressure, their interaction with salt overload during pregnancy, and how these mechanisms remain intact in the presence of salt overload and Mg-gluconate (Table 3). As previously suggested (Beauséjour et al., 2003; Auger et al., 2004), salt overload blocks, in pregnant rats, the decreased sensitivity to vasoconstrictors characteristic of rat pregnancy during the last week of gestation, and, in turn, Mg-gluconate would prevent this decreased sensitivity to vasoconstrictors. This hypothesis needs to be tested.

The effect of sodium supplementation in pregnant rats on the level of lipid peroxidation and thus concomitantly on PMCA activity opens new possibilities for the interpretation of the effect of the presence of Mg-gluconate in this animal model of PE. In this regard, there is an inversely proportional relationship between lipid peroxidation levels and PMCA activity (Matteo et al., 1998; Abad et al., 2012). Thus, the role played by Mg-gluconate as an antioxidant could shed light on its effect on blood pressure in salt-loaded pregnant rats. The role of PMCA in adjusting the fine intracellular Ca^2+^ concentration ([Ca^2+^]_i_) is well known (Carafoli, 1994; Brini and Carafoli, 2011). In mammalian tissues, the cytoplasmic Ca^2+^ concentration is maintained at approximately 100 nm primarily due to the activities of PMCA and smooth endoplasmic reticulum Ca^2+^-ATPase (SERCA; Clapham, 2007). In these tissues, the different PMCA isoforms (PMCA1-PMCA4) are known to be encoded by at least four distinct genes (ATP2B1-ATP2B4). Of these PMCA isoforms, PMCA1 and PMCA4 are ubiquitinated, whereas PMCA2 and PMCA3 are specifically abundant in excitable cells (Prasad et al., 2007). The control of [Ca^2+^]_i_ is critical for normal vascular contraction because an increase in [Ca^2+^]_i_ can initiate smooth muscle contraction and thereby increase total peripheral resistance (Filo et al., 1965; Somlyo and Somlyo, 1994). In this particular, it has been found that the [Ca^2+^]_i_ of spontaneously hypertensive rats is higher than that of normotensive rats, such as Wistar-Kyoto (Sugiyama et al., 1990). In these animals, it has been shown that both basal and angiotensin II-induced Ca^2+^ uptake are significantly increased in vascular smooth muscle cells of spontaneously hypertensive rats, which is accompanied by an increase in [Ca^2+^]_i_ (Orlov et al., 1993; Chen and Roufogalis, 1994; Samain et al., 1999). The response of [Ca^2+^]_i_ control mechanisms is given by an increase in ATP2B1 gene expression and with it the level of PMCA1 mRNA (Monteith et al., 1997). Therefore, inhibition of PMCA in salt-loaded pregnant rats as a consequence of increased lipid peroxidation could lead to increased [Ca^2+^]_i_ levels and explain, at least in part, the increase in blood pressure. Mg-gluconate, by preventing the increase in lipid peroxidation associated with salt overload in rats, would prevent the decrease in PMCA activity and thus prevent the increase in [Ca^2+^]_i_. There is no doubt that this possibility requires further studies.

The presence of proteinuria in salt-loaded pregnant rats (Table 3) is an indication of renal dysfunction. In this regard, the increased oxidative stress associated with sodium supplementation could likely be producing and important damage in the glomerular membrane. The effect of Mg-gluconate on the protein excretion in salt-loaded pregnant rats is very important although we do not know the precise molecular mechanisms involved in the apparent blockade of the deleterious effect of the sodium supplementation on the renal function produced by the presence of Mg-gluconate (Table 3). We can speculate that the antioxidant effect of Mg-gluconate shown in this study (Figures 2A–C) could be avoiding the damage of the glomerular membrane and therefore reducing the level of proteinuria.

A variety of Mg^2+^ forms in addition to MgSO_4_ is available, i.e., MgO, Mg(OH)_2_, Mg-citrate, Mg-aspartate, Mg-gluconate, Mg-lactate, Mg-stearate, and MgCl_2_. The use of oral Mg^2+^ salts in pregnancy and particularly in PE has been tested in several studies (Makrides et al., 2014). A large well-designed doubleblind randomized trial to verify the effect of oral magnesium supplementation with Mg-citrate on PE incidence in low-income pregnant women was carried out in Brazil (De Araújo et al., 2020a,b). The authors concluded that oral magnesium supplementation during pregnancy is yet not proven to be effective in preventing PE among low-income and low-risk pregnant women. What we are suggesting is that Mg-gluconate seems to be a better antioxidant than most of the available Mg salts. From a theoretical point of view, Mg-gluconate has a higher antioxidant capacity mainly due to the presence of 10 hydroxyl groups in the two anions of this salt. In fact, the treatment of the salt-loaded pregnant rats with Mg-gluconate avoids the rise in the level of lipid peroxidation (Figures 2A–C).

It is well known that Mg exists complexed to multiple molecules intracellularly and it seems that the intracellular Mg concentration is the one that is relevant. However, Kisters et al. (2000) observed a lower content of Mg^2+^ and a higher content of Ca^2+^ in membranes of red blood cells from preeclamptic pregnant women, as compared with the red blood cells of uncomplicated pregnant women. These alterations could lead to interactions of these ions with membrane components, resulting in modifications of the lipid microenvironment that interacts with membrane transporters. This group found no differences between blood plasma magnesium concentrations in the healthy pregnant group and the preeclamptic group. Furthermore, there was no difference in intracellular magnesium concentration, at least for the red blood cells, for both healthy pregnant group and the preeclamptic group. These authors suggested that plasma membrane magnesium concentrations in PE may contribute to the development in hypertension in pregnancy. In fact, Chiarello et al. (2014) found that a minimum concentration of Mg^2+^ is required to protect the membranes and to circumvent the rise of lipid peroxidation of the syncytiotrophoblast plasma membranes seen during PE. In this regard, elevated serum Mg^2+^ concentrations may contribute to the maintenance of minimal Mg^2+^ contents in plasma membranes of the animal model of PE (Supplementary Table 2).

## Conclusion

The treatment of salt-loaded pregnant rats with Mg-gluconate avoids the rise in the level of lipid peroxidation and the concomitant lowering of the PMCA activity of their red blood cell membranes, which are associated with sodium supplementation in these animals, reaching values similar to those from control pregnant rats. Also, the presence of Mg-gluconate during sodium supplementation of the pregnant rats avoids the rise of the osmotic fragility of their red blood cells, keeping values similar to those from control pregnant rats. Mg-gluconate seems to be an important candidate for the replacement of the MgSO_4_ therapy of preeclamptic women.

## Data Availability Statement

The raw data supporting the conclusions of this article will be made available by the authors, without undue reservation.

## Ethics Statement

The animal study was reviewed and approved by Animal ethics committee (Cobianim) Instituto Venezolano de Investigaciones Científicas, IVIC.

## Author Contributions

DR, CA, FP, and RM conceived the experiments and analyzed the data. DR, SP, YM, and DC carried out the experiments. All authors were involved in writing the paper and had final approval of the submitted version.

## Funding

This work was supported by the Fondo Nacional de Ciencia, Tecnología e Innovación (FONACIT) [project F-2005000222], Venezuela.

## Conflict of Interest

The authors declare that the research was conducted in the absence of any commercial or financial relationships that could be construed as a potential conflict of interest.

## Publisher’s Note

All claims expressed in this article are solely those of the authors and do not necessarily represent those of their affiliated organizations, or those of the publisher, the editors and the reviewers. Any product that may be evaluated in this article, or claim that may be made by its manufacturer, is not guaranteed or endorsed by the publisher.

## Supplementary Material

Supplementary Figure 1 **(A)** Effect of treatment with Mg-gluconate on the osmotic lysis curves of intact red blood cells from control pregnant rats. Pregnant female Sprague–Dawley rats (bodyweight 225–250 g, 3 months old, CP) had tap water during the last week of their pregnancy, with and without Mg-gluconate in the drinking solution. The average daily Mg-gluconate intake (g/kg b.d.) was similar to that shown in Supplementary Table 2. **(B)** Effect of the treatment with Mg-gluconate on the osmotic lysis curves of intact red blood cells from salt-loaded pregnant rats. Salt-loaded pregnant (bodyweight 225–250 g, 3 months old, SLP) rats were kept drinking a solution of 1.8% NaCl, with and without Mg-gluconate during the last week of their pregnancy. The average daily Mg-gluconate intake (g/kg b.d.) was similar to that shown in Supplementary Table 2. OS_50_ is defined as the osmolar concentration that produces 50% hemolysis of the added red blood cells. The lysis was performed as indicated in the Materials and Methods section. Values are means ± S.E. for *n* = 8.
