# Supplementary figures and images for: Effect of Mg-Gluconate on the Osmotic Fragility of Red Blood Cells, Lipid Peroxidation, and Ca2+-ATPase (PMCA) Activity of Placental Homogenates and Red Blood Cell Ghosts From Salt-Loaded Pregnant Rats
Source: Front Physiol. 2022 Jan 27;13:794572. doi: 10.3389/fphys.2022.794572 (PMC8829449; doi:10.3389/fphys.2022.794572)

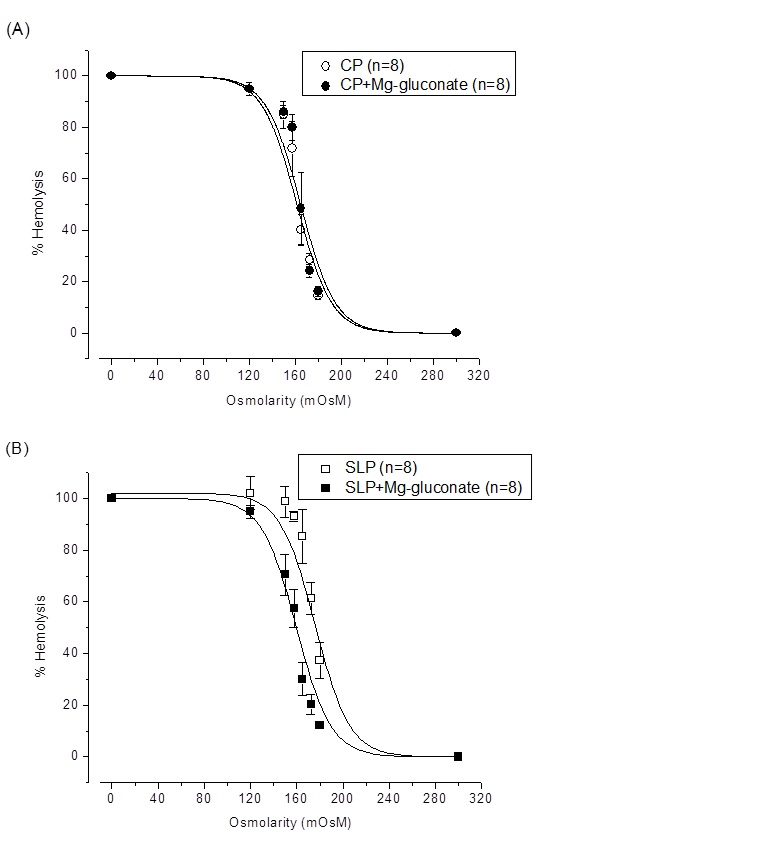

Supplement: Supplementary Figure 1 — (A) Effect of treatment with Mg-gluconate on the osmotic lysis curves of intact red blood cells from control pregnant rats. Pregnant female Sprague–Dawley rats (bodyweight 225–250 g, 3 months old, CP) had tap water during the last week of their pregnancy, with and without Mg-gluconate in the drinking solution. The average daily Mg-gluconate intake (g/kg b.d.) was similar to that shown in Supplementary Table 2. (B) Effect of the treatment with Mg-gluconate on the osmotic lysis curves of intact red blood cells from salt-loaded pregnant rats. Salt-loaded pregnant (bodyweight 225–250 g, 3 months old, SLP) rats were kept drinking a solution of 1.8% NaCl, with and without Mg-gluconate during the last week of their pregnancy. The average daily Mg-gluconate intake (g/kg b.d.) was similar to that shown in Supplementary Table 2. OS50 is defined as the osmolar concentration that produces 50% hemolysis of the added red blood cells. The lysis was performed as indicated in the Materials and Methods section. Values are means ± S.E. for n = 8. [file Image_1.JPEG]
