# Supplementary material for: Effect of Mg-Gluconate on the Osmotic Fragility of Red Blood Cells, Lipid Peroxidation, and Ca2+-ATPase (PMCA) Activity of Placental Homogenates and Red Blood Cell Ghosts From Salt-Loaded Pregnant Rats
Source: Front Physiol. 2022 Jan 27;13:794572. doi: 10.3389/fphys.2022.794572 (PMC8829449; doi:10.3389/fphys.2022.794572)
Supplement: Supplementary file 2 [file Table_1.docx]

**Supplementary Table 1**

**Effect of the treatment with Mg-gluconate on the serum Mg^2+^ levels of control (CNP) and salt-loaded non-pregnant rats (SLNP)**

| **Average daily Mg-gluconate intake**  **(g/kg b.d.)** | **Serum Mg^2+^ levels (mg/ml)** | | | | | |
| --- | --- | --- | --- | --- | --- | --- |
|  | **CNP** | **P** | **n** | **SLNP** | **P** | **n** |
| 0 | 0.024±0.001 |  | 4 | 0.021±0.003 |  | 8 |
| 0.59±0.03 | 0.025±0.003 | ns | 4 | 0.025±0.002 | ns | 4 |
| 1.27±0.05 | 0.024±0.001 | ns | 3 | 0.023±0.002 | ns | 11 |
| 1.97±0.09 | 0.028±0.001 | <0.05 | 6 | 0.029±0.001 | <0.05 | 6 |
| 2.70±0.13 | 0.031±0.001 | <0.01 | 3 | 0.029±0.002 | <0.05 | 8 |

Non-pregnant female Sprague-Dawley rats (bodyweight 225–250 g, three months old, CNP) had tap water during one week, with and without Mg-gluconate in the drinking solution. Salt-loaded non-pregnant (bodyweight 225–250 g, 3 months old, SLNP) rats were kept drinking a solution of 1.8% NaCl, with and without Mg-gluconate for one week. Values are expressed as mean±S.E. P-value for comparison between groups with and without Mg-gluconate (t-test).
