# Supplementary material for: Effect of Mg-Gluconate on the Osmotic Fragility of Red Blood Cells, Lipid Peroxidation, and Ca2+-ATPase (PMCA) Activity of Placental Homogenates and Red Blood Cell Ghosts From Salt-Loaded Pregnant Rats
Source: Front Physiol. 2022 Jan 27;13:794572. doi: 10.3389/fphys.2022.794572 (PMC8829449; doi:10.3389/fphys.2022.794572)
Supplement: Supplementary file 3 [file Table_2.docx]

**Supplementary Table 2**

**Effect of the treatment with Mg-gluconate on the serum Mg^2+^ levels of control (CP) and salt-loaded pregnant rats (SLP)**

| **Animal Group** | **Average daily Mg-gluconate intake**  **(g/kg b.d.)** | **Serum Mg^2+^ levels (mg/ml)** | **n** |
| --- | --- | --- | --- |
| CP | 0 | 0.022±0.002 | 5 |
|  | 2.87±0.03 | 0.030±0.002^(a)^ | 4 |
| SLP | 0 | 0.022±0.001 | 6 |
|  | 2.79±0.06 | 0.031±0.001^(b)^ | 4 |

Pregnant female Sprague-Dawley rats (bodyweight 225–250 g, 3 months old, CP) had tap water during the last week of their pregnancy, with and without Mg-gluconate in the drinking solution. Salt-loaded pregnant (bodyweight 225–250 g, 3 months old, SLP) rats were kept drinking a solution of 1.8% NaCl, with and without Mg-gluconate during the last week of their pregnancy. Values are expressed as mean±S.E. Comparisons between treatment conditions were assessed by one-way ANOVA with the post hoc analysis with the Student–Newman–Keuls test. P-value for the ANOVA test was <0.001.

1. p< 0.01 vs CP
2. p<0.01 vs SLP
