# Supplementary material for: Effect of Mg-Gluconate on the Osmotic Fragility of Red Blood Cells, Lipid Peroxidation, and Ca2+-ATPase (PMCA) Activity of Placental Homogenates and Red Blood Cell Ghosts From Salt-Loaded Pregnant Rats
Source: Front Physiol. 2022 Jan 27;13:794572. doi: 10.3389/fphys.2022.794572 (PMC8829449; doi:10.3389/fphys.2022.794572)
Supplement: Supplementary file 4 [file Table_3.docx]

**Supplementary Table 3**

**Effects of the treatment with Mg-gluconate on water intake, food intake, sodium intake, magnesium intake, and weight gain in control (CP) and salt-loaded pregnant rats (SLP)**

| **Parameter** | **CP** | **CP+Mg-gluconate** | **SLP** | **SLP+Mg-gluconate** |
| --- | --- | --- | --- | --- |
| Water intake (ml) | 306.08±8.32 (6) | 320.18±6.79 (6) | 488.50±24.19 (7)^(a)^ | 314.76±9.76 (7) |
| Food intake (g) | 152.23±3.05 (6) | 172.33±9.82 (6) | 104.69±11.53 (7)^(a)^ | 167.00±4.69 (7) |
| Sodium intake (mg) | 170.00±2.00 (6) | 190.00±4.00 (6) | 8,980±380 (8)^(a)^ | 5,850±460 (7)^(b)^ |
| Magnesium Intake (g) | 0.15±0.01 (6) | 5,912.00±475.00 (6)^(c)^ | 0.10±0.01 (8) | 5,455.95±530.35 (7)^(d)^ |
| Weight variation 15-22 (g) | 69.00±7.50 (15) | 47.00±6.04 (9)^(c)^ | 24.00±6.96 (14)^(c)^ | 8.00±3.83 (12)^(e)^ |

The average daily Mg-gluconate intake (g/kg b.d.) was similar to that shown in Supplementary Table II. Values in parentheses indicate the total number of mothers processed in each case. Values expressed as the mean±S.E. Comparisons between treatment conditions were assessed by one-way ANOVA with the post hoc analysis with the Student–Newman–Keuls test. In each row, P-value for the ANOVA test was <0.001.

1. p< 0.001 vs CP, vs SLP+Mg-gluconate
2. p<0.001 vs CP+Mg-gluconate
3. p< 0.001 vs CP
4. p<0.001 vs SLP
5. p< 0.01 vs CP+Mg-gluconate
